# Editorial: Immune Modulation by Flavonoids

**DOI:** 10.3389/fimmu.2022.899577

**Published:** 2022-04-11

**Authors:** David W. Hoskin, Melanie R. Power Coombs

**Affiliations:** ^1^ Department of Pathology, Dalhousie University, Halifax, NS, Canada; ^2^ Biology Department, Acadia University, Wolfville, NS, Canada

**Keywords:** flavonoid, inflammation, phytochemical, polyphenol, microbiota

Flavonoids are polyphenolic phytochemicals that are secondary metabolites from plants. In the past, flavonoids have been shown to modulate immune responses such as promoting anti-cancer T cell responses (1), reducing reactive oxygen species (ROS) production (2), and inhibiting inflammatory processes (3, 4). Given the increasing use of flavonoids as nutraceuticals, there is an urgent need for more research to clarify the role of flavonoids and their mechanism of action in the modulation of immune responses. This collection of articles consists of several research papers and a review article that examine the impact of phytochemicals on cellular processes and their role in limiting inflammation (**Figure 1**).

**Figure 1:**
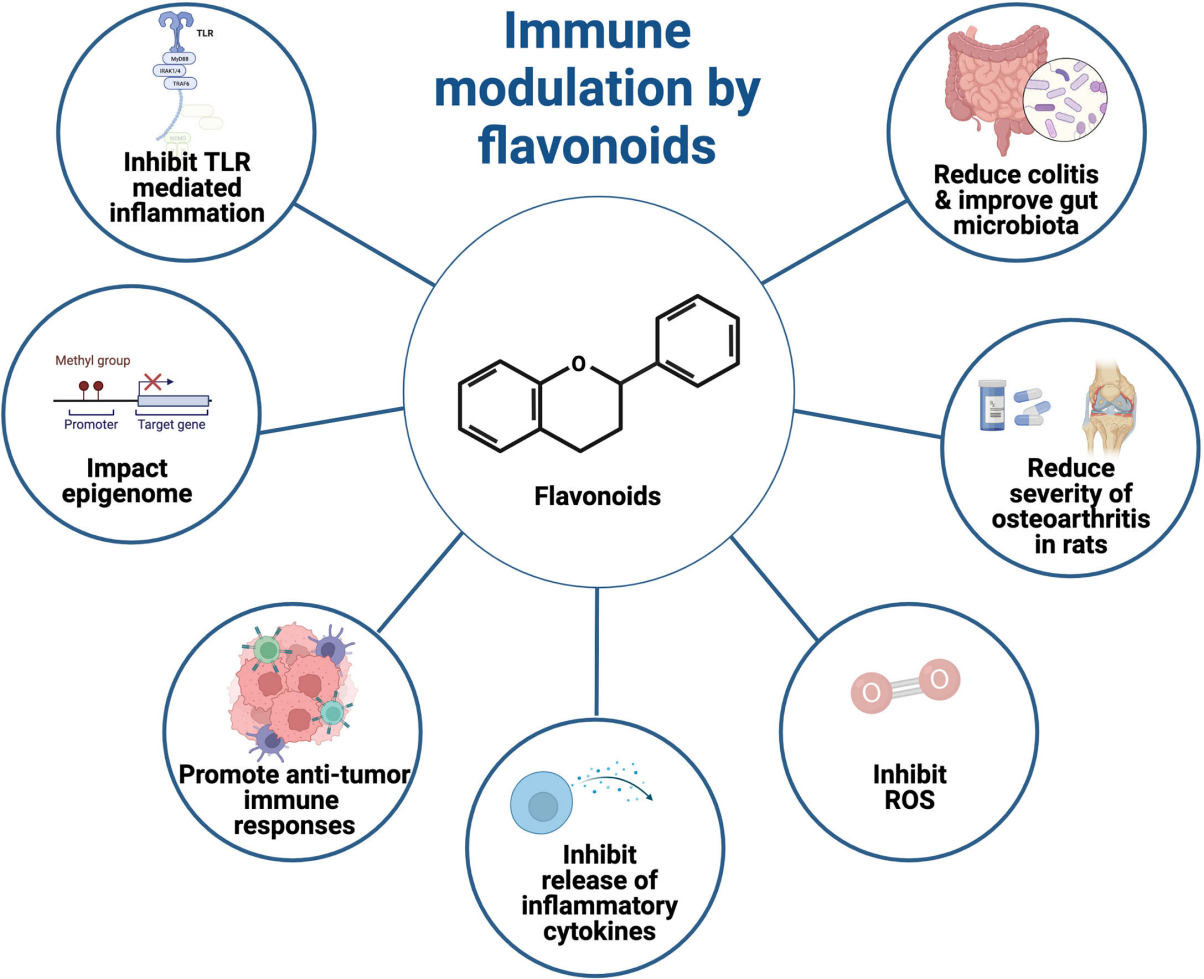
Flavonoids that modulate immune responses may have beneficial therapeutic potential. Image created using biorender.com.

In their review article, Saleh et al. discuss the effect of several different phytochemicals on multiple components of the Toll-like receptor (TLR)-4 signaling pathway. The impact of these phytochemicals on epigenetic modifications such as the methylation and acetylation of genes related to NF-κB-regulated inflammatory gene expression, as well as on microRNAs involved in inflammation are highlighted.

Studies conducted using animal models of inflammation have revealed the therapeutic potential of natural products such as curcumin, kaempferol, and mogroside V for the prevention of different inflammatory diseases (Yabas et al., Qu et al., and Dou et al.). Curcumin, which is a polyphenol derived from the commonly used spice turmeric (*Curcuma longa*), is known to have biological activities that include anti-inflammatory, antioxidant, and antiproliferative effects (5). In this collection of research articles, Yabas et al. report on an animal study that used a novel curcumin formulation called Next Generation Ultrasol Curcumin (NGUC), which exhibits substantially improved oral bioavailability. In a rat model of osteoarthritis, oral administration of NGUC reduced disease severity by modulating inflammatory mediators and oxidative stress markers.

Research by Qu et al. focuses on the anti-inflammatory and antioxidant activities of kaempferol, a medicinal plant-derived flavonoid that decreases inflammatory bowel disease in a dextran sulfate sodium (DSS)-induced model of colitis in mice. Their investigation of the mechanism of action of kaempferol indicates that administration of this phytochemical improves gut microbiota and decreases inflammatory mediators, thereby contributing to the beneficial effect of kaempferol on colitis. A fecal microbiota transplant from kaempferol-treated mice to mice with colitis confirmed the effects of kaempferol on modulating the gut microbiota. Kaempferol may exert protective effects against colitis by regulating the gut microbiota and modulating TLR4-related signaling pathways associated with inflammation.

Research by Dou et al. elucidates the impact of the flavonoid mogroside V on inflammatory gene and protein expression associated with ovalbumin (OVA)-induced lung inflammation in mice. The authors identify NF-κB and JAK-STAT signaling pathways as key targets of inhibitory mogroside V in OVA-induced mice, resulting in reduced expression of immunoglobulin E, interleukin-5, and tumor necrosis factor-α. These effects may account for the beneficial effect of mogroside V-containing *Siraitia grosvnorii* on pulmonary inflammation.

Our understanding of the effects of flavonoids on biological processes is constantly improving. Flavonoids impact immune responses to pathogens and other antigens, regulate intestinal mucosal immune responses, and modulate anti-tumor immunity. The consumption of flavonoid-rich fruits and vegetables may therefore improve immune function; however, the mechanism of action of flavonoids in humans need to be explored further. Given the increasing prevalence of chronic inflammatory diseases, flavonoids may yield potential therapeutic agents.

## Author Contributions

MC and DH wrote the editorial. MC made **Figure 1**. All authors contributed to the article and approved the submitted version.

## Conflict of Interest

The authors declare that the research was conducted in the absence of any commercial or financial relationships that could be construed as a potential conflict of interest.

## Publisher’s Note

All claims expressed in this article are solely those of the authors and do not necessarily represent those of their affiliated organizations, or those of the publisher, the editors and the reviewers. Any product that may be evaluated in this article, or claim that may be made by its manufacturer, is not guaranteed or endorsed by the publisher.

## References

[1] Coombs MRP Harrison ME Hoskin DW. Apigenin Inhibits the Inducible Expression of Programmed Death Ligand 1 by Human and Mouse Mammary Carcinoma Cells. Cancer Lett (2016) 380:424–33. doi: 10.1016/j.canlet.2016.06.023 27378243

[2] Mishra A Sharma AK Kumar S Saxena AK Pandey AK. Bauhinia Variegata Leaf Extracts Exhibit Considerable Antibacterial, Antioxidant, and Anticancer Activities. BioMed Res Int (2013) 2013:915436. doi: 10.1155/2013/915436 24093108 PMC3777169

[3] Bernard M Furlong SJ Power Coombs MR Hoskin DW. Differential Inhibition of T Lymphocyte Proliferation and Cytokine Synthesis by [6]-Gingerol, [8]-Gingerol, and [10]-Gingerol. Phytother Res (2015) 29:1707–13. doi: 10.1002/ptr.5414 26178781

[4] Yahfoufi N Alsadi N Jambi M Matar C. The Immunomodulatory and Anti-Inflammatory Role of Polyphenols. Nutrients (2018) 10:E1618. doi: 10.3390/nu10111618 30400131 PMC6266803

[5] Salehi B Stojanović-Radić Z Matejić J Sharifi-Rad M Anil Kumar NV Martins N. The Therapeutic Potential of Curcumin: A Review of Clinical Trials. Eur J Med Chem (2019) 163:527–45. doi: 10.1016/j.ejmech.2018.12.016 30553144

